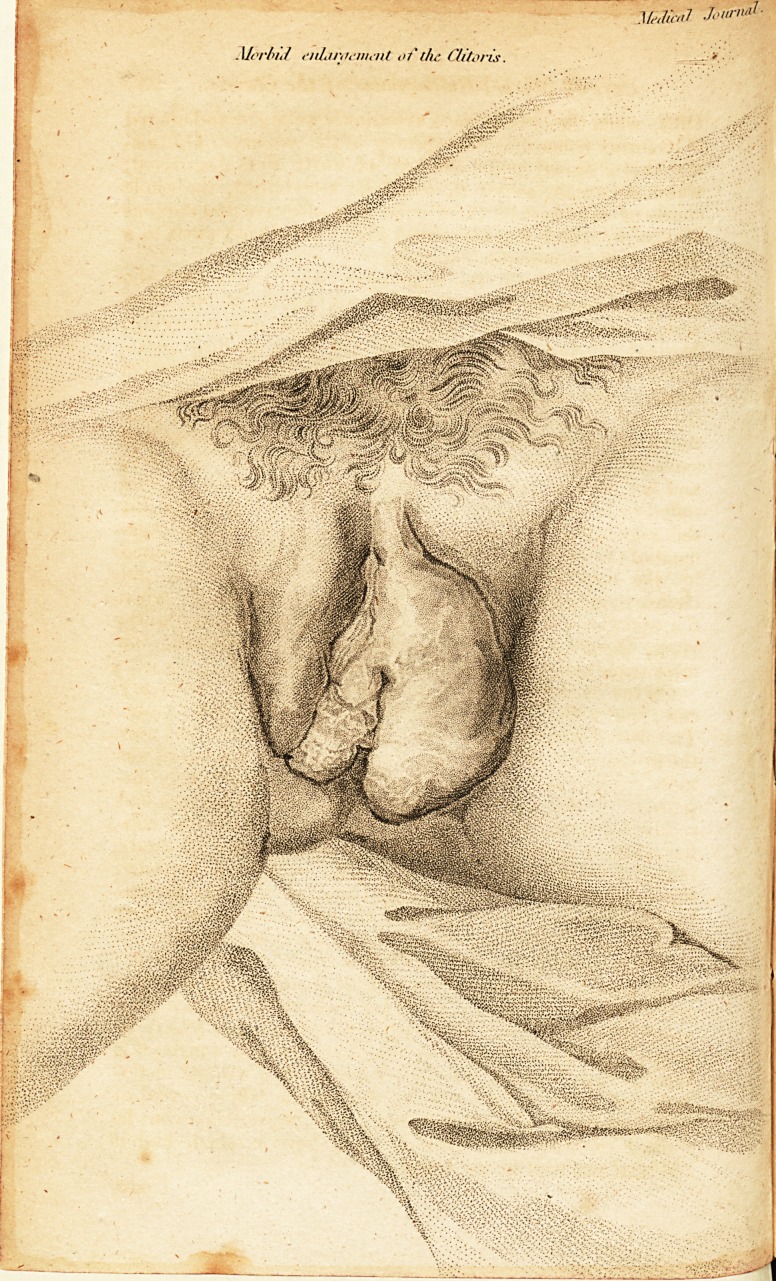# Case of Morbid Enlargement of the Clitoris

**Published:** 1811-03

**Authors:** W. M. Lewis


*To the Editors of the Medical and Physical Journal.*

### *Case of Morbid Enlargement of the Clitoris.*

*(With an Engraving.)*

GENTLEMEN,

THE subject of this case is a female about twenty-seven years of age, has been twice infected with syphilis, the periods of which I have satisfactorily ascertained, in order that the rapid progress of the morbid enlargement of the *Clitoris* might be estimated. I first saw her on the 5th of March,

Morbid enlargement of the Clitoris.

1810, when the clitoris was exceedingly enlarged and altered in its form, concealing the opening of the vagina. On raising this diseased mass, clusters of warts were discovered, which occupied, as far as could be ascertained, the whole course of the vagina: there was a most offensive and copious discharge, which had excoriated the neighbouring parts to a degree that prevented her walking.

The account she at this time gave, was simply that she had contracted the venereal disease six months before, from which time the *clitoris* had been progressively enlarging. She had, during this period, refrained from medical assistance, nor had she used, directly or indirectly, any curative means. She was immediately put on a course of mercurial friction; and at the expiration of six weeks the general character of the disease was much amended, the discharge had subsided, the warts had yielded to the influence of mercury, and her general health was considerably improved. I now stated to her the propriety of submitting to an operation for the removal of the tumour formed by the morbid enlargement of the clitoris, and which in no degree had been lessened by the mercurial course. To this, however, she did not choose to submit. Thus ended my first attendance; when the specific was supposed to have destroyed the syphilitic virus, though *sequela* of its depredations still remained; the most remarkable of which was the diseased clitoris.

On the 15th of December last, she again came under my care, and with more aggravated circumstances than before. The altered structure and enlargement of the clitoris had now the appearance shewn in the Engraving; the discharge was more copious and offensive; and her health was seriously impaired. I was particular in my inquiries whether she had received a fresh infection, and by those inquiries I was confirmed in an opinion suggested by the symptoms: no doubt indeed remained of her having again contracted syphilis. She underwent a second course of mercury, the same favorable change was experienced, with the exception of the warts in the vagina remaining both stationary and numerous.

When it was presumed that the mercurial action had destroyed the syphilitic, and her general health was in a proper state, it was again proposed to her to have this inconvenient and distressing tumour removed by an operation. It may be proper to observe, that under these two mercurial courses this tumour had remained unreduced in size, and without melioration of its condition: indeed, during the whole period of its existence, it rapidly increased, and no estimate can be formed of what magnitude it might have reached, had it not been removed by the knife.

Under

Under these circumstances the patient was induced to consent to the operation ; and on the 24th of January last, the diseased mass was removed by Mr. Brooks *at his Theatre during lecture*. Some hemorrhage took place on the division of the parts, rendering an application of ligature necessary to several vessels. There was very little subsequent inflammation or symptomatic fever, and the wound is nearly healed.

W. M. LEWIS.

*Feb.* 12, 1811.

## Figures and Tables

**Figure f1:**